# Immunohistochemistry in the pathologic diagnosis and management of thyroid neoplasms

**DOI:** 10.3389/fendo.2023.1198099

**Published:** 2023-05-31

**Authors:** Anna Crescenzi, Zubair Baloch

**Affiliations:** ^1^ Pathology, University Campus Bio-Medico of Rome, Fondazione Policlinico, Rome, Italy; ^2^ Pathology & Laboratory Medicine, University of Pennsylvania Medical Center, Perelman School of Medicine, Philadelphia, PA, United States

**Keywords:** thyroid, pathology, immunohistochemistry, molecular, biomarkers, cytology

## Abstract

The use of immunohistochemistry cannot be underestimated in the everyday practice of thyroid pathology. It has evolved over the years beyond the traditional confirmation of thyroid origin to molecular profiling and the prediction of clinical behavior. In addition, immunohistochemistry has served to implement changes in the current thyroid tumor classification scheme. It is prudent to perform a panel of immunostains, and the immunoprofile should be interpreted in light of the cytologic and architectural features. Immunohistochemistry can also be easily performed in the limited cellularity specimen preparation generated from thyroid fine-needle aspiration and core biopsy; however, it will require laboratory validation of immunostains specific to these preparations to avoid diagnostic pitfalls. This review discusses the application of immunohistochemistry in thyroid pathology with a focus on limited cellularity preparations.

## 1 Introduction

Thyroid carcinoma is the most common malignancy of endocrine organs and accounts for approximately 1% of all cancers. As per the new WHO classification scheme, the neoplasms of the thyroid gland are stratified into the following main categories: follicular cell-derived neoplasms, C-cell derived neoplasms, mixed medullary and follicular cell-derived neoplasms, salivary gland type carcinomas, thyroid tumors of uncertain histogenesis, thymic tumors within the thyroid, and embryonal thyroid neoplasms. Even though the majority of thyroid neoplasms can be diagnosed on the basis of cellular and architectural features, difficulties in the diagnosis can occur due to overlapping histomorphologic features between primary and secondary thyroid neoplasms and partial or complete loss of differentiation (1).

The well-differentiated thyroid carcinomas originating from the thyroid follicular cells show either follicular or papillary growth patterns or an admixture of both. The presence of colloid within follicles and complex papillary structures with diagnostic nuclear cytology in these neoplasms facilitate the diagnosis of these neoplasms (1–6). The solid and “insular” growth pattern of poorly differentiated carcinoma, especially in cases with an inconspicuous or lack of a well-differentiated component, can be mistaken for C-cell-derived medullary thyroid carcinoma or metastatic neuroendocrine neoplasm arising at other body sites. Anaplastic/undifferentiated carcinoma can show varying cytologic features and growth patterns mimicking lymphoma, mesenchymal tumors, and secondary tumors of the thyroid gland (2, 4).

In the abovementioned scenarios, employing a panel of immunostains can help solve diagnostic conundrums. In addition, immunohistochemistry (IHC) has proven to be helpful in the diagnosis of the following rare tumors: mixed follicular and medullary thyroid carcinoma, salivary gland type carcinomas, tumors of uncertain histogenesis, and intra-thyroidal thymic neoplasms (2). Employing mutation-specific antibodies can serve to distinguish between papillary carcinomas harboring the BRAFV600E mutation from RAS-like neoplasms. The utility of proliferation markers such as Ki67 cannot be underestimated in the grading of thyroid carcinoma, which has been shown to be a predictor of clinical behavior in both follicular and C-cell-derived neoplasms (1, 2, 7).

## 2 Immunochemistry: basic concepts

Immunostaining is an easy, cheap, and widely available technique for selectively identifying specific molecules in tissue sections and cytological preparations. This technique is based on the use of antibodies (also called immunoglobulins) that are Y‐shaped globular proteins formed by two light chains and two heavy chains, held together by disulphide bonds (8). The molecular recognition abilities of the antibodies allow for various applications in diagnostic pathology. Each antibody is capable of binding only to a specific antigen (9); therefore, they are currently applied in pathology to identify the cell lineage, examine the expression of biomarkers, characterise tumors, and more recently, determine the expression of targets for tailored therapies. For diagnostic purposes, antibodies are labelled, directly or with a multistep chain, with a visible molecule that allows the recognition of their binding reaction in tissue sections or cytological samples. The immunostaining protocol is mostly automated in many laboratories, which improves the reproducibility of the reaction product, although this standardization is usually developed for IHC on histological sections, whereas dedicated recommendations and practice paradigms are still lacking for cytological samples. This is probably because of the large variability in cellular preparations (conventional smears, thin layer cytology, and cell-blocks), different treatment of the specimens for immunostaining, and interpretative cutoffs (7, 8, 10–13).

As stated above, there are three main reasons for the application of immunocytochemistry in thyroid pathology: ***determining cell and site of origin, differentiating benign from malignant neoplasms, and influencing clinical management.***

## 3 Determining cell and site of origin

IHC is an indispensable tool that complements routine histologic techniques for elucidating differential diagnosis in histologic and cytologic preparations. The use of IHC in thyroid pathology is based on knowledge regarding cell of origin and further characterization. It is mainly suggested for lesions that are suspected of non-follicular or non-thyroidal origin (e.g., parathyroid, medullary thyroid carcinoma, lymphoma, metastases from other organs—secondary tumors, etc.) (14, 15).

### 3.1 Thyroid follicular cell lineage markers

#### 3.1.1 Thyroid follicular cell origin

**Figure 1:**
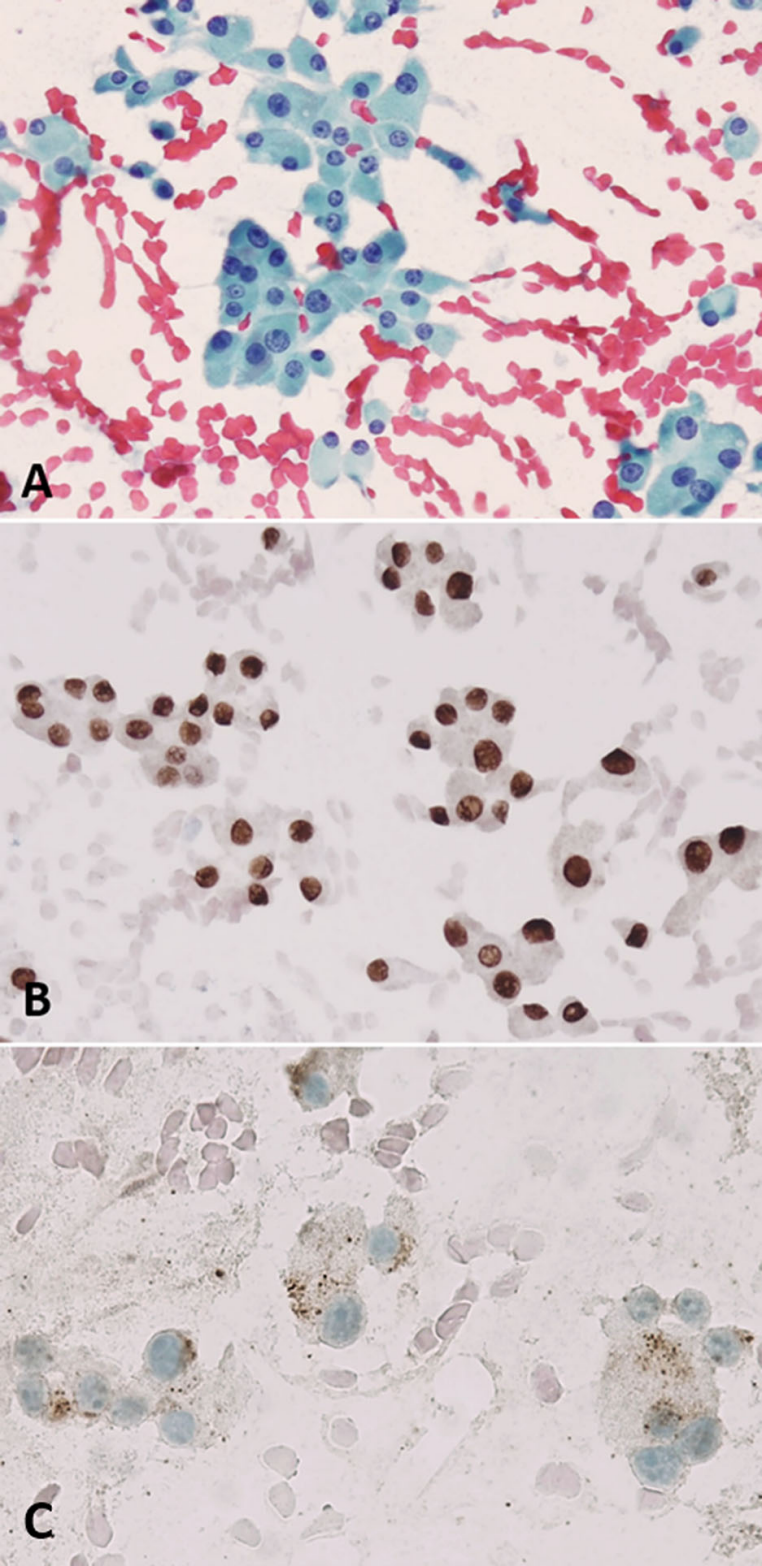
An oncocytic proliferative lesion, direct smear. **(A)** Papanicolaou stain shows numerous oncocytic cells with variable nuclear dimensions. **(B)** PAX8 immunostaining performed on a destained slide shows strong nuclear positivity in the cells. **(C)** Thyroglobulin immunostaining performed on a destained slide shows a punctate dot-like positive cytoplasmic reaction.

**Figure 2:**
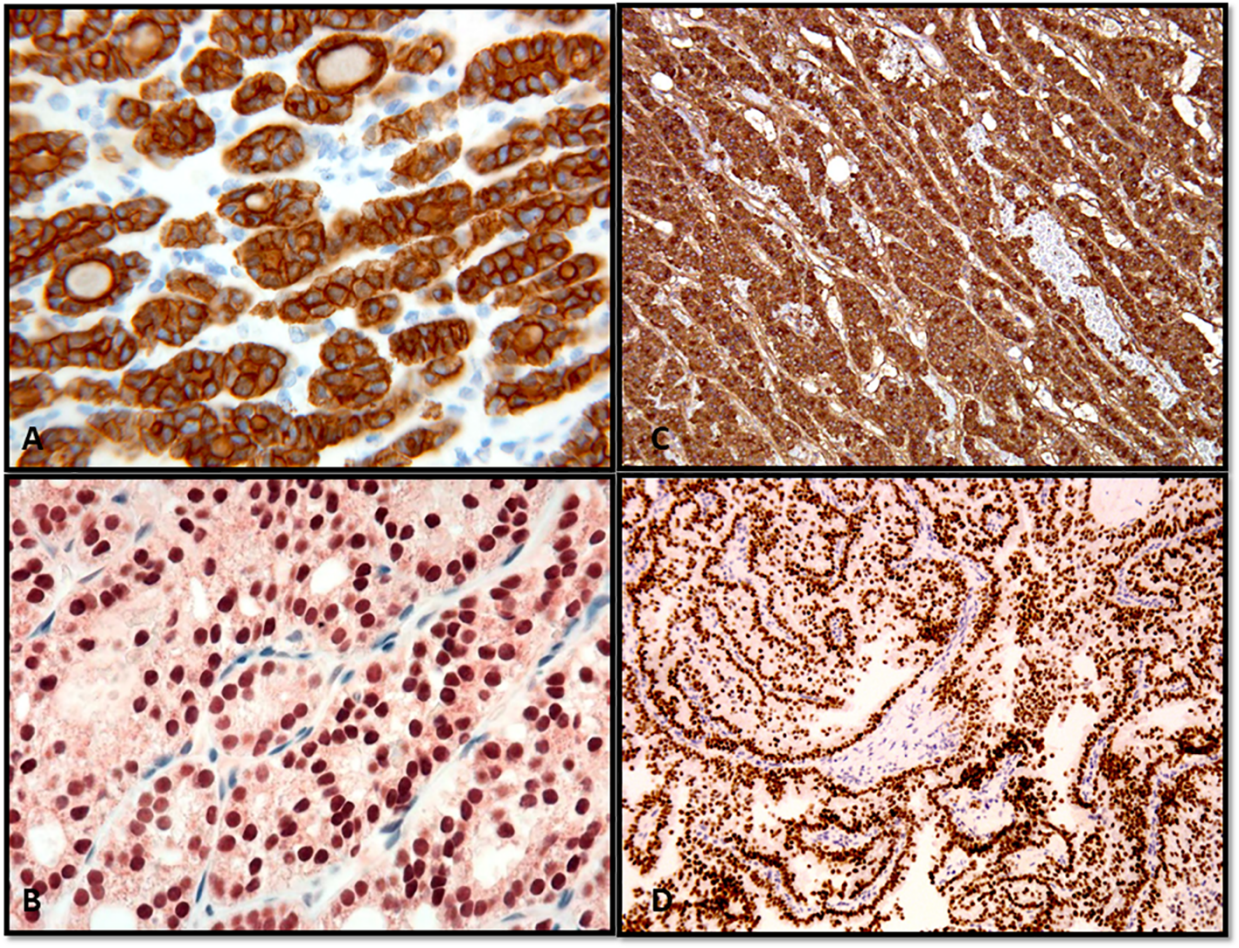
Confirmation of the thyroid follicular cell origin through the expression of thyroglobulin and TTF in follicular adenoma **(A)**, thyroglobulin; **(B)**, TTF-1) and papillary thyroid carcinoma **(C)**, thyroglobulin; **(D)**, TTF-1).

Is usually confirmed by a panel of immunohistochemical markers that can identify metastasis to the thyroid gland from other organs, thyroid cancer metastasis to extra-thyroidal sites, and thyroid carcinoma arising in ectopic thyroid tissue. The most important markers of thyroid follicular cell derivation are thyroglobulin (TG), thyroid transcription factor 1 (TTF1), and paired-box gene 8 (PAX8); antibodies against these are often used in a panel to overcome the limits of a single antibody (**Figures 1**, **2**). It is relevant to know some details about these antibodies (7, 13, 16–18).

#### 3.1.2 Thyroglobulin

**Figure 3:**
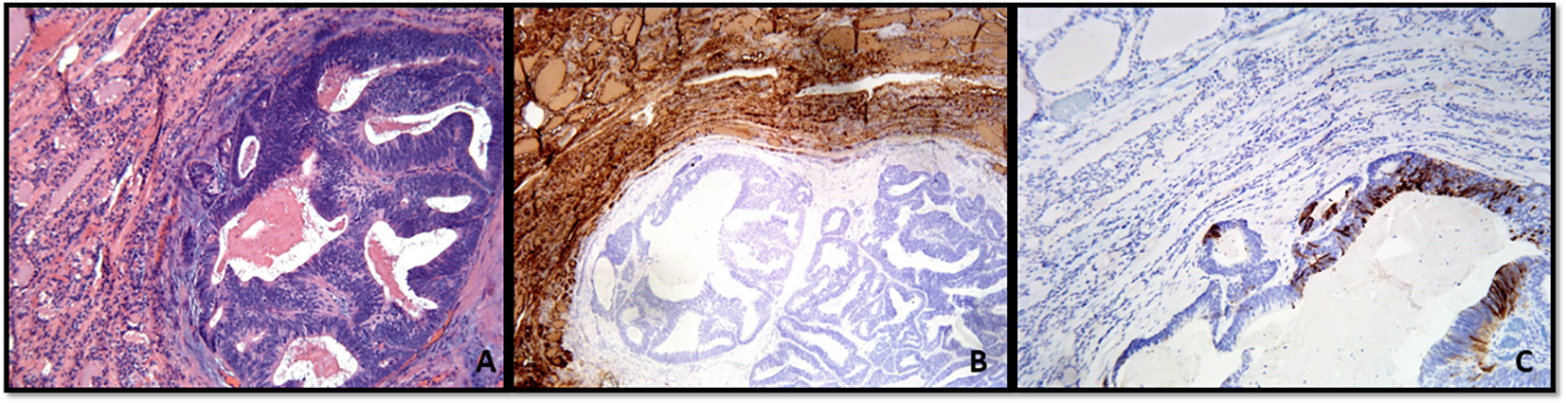
**(A–C)** Metastatic colonic adenocarcinoma to the thyroid gland showing the neoplastic gland in the background thyroid parenchyma **(A)**, Hematoxylin and Eosin stain). **(B, C)** With immunohistochemistry, thyroglobulin expression is only present in the thyroid parenchyma **(B)**, and the tumor shows cytokeratin 20 expression **(C)**.

Is the most specific marker of thyroid follicular cell derivation. It is a glycoprotein manufactured by thyrocytes, from which it is secreted into thyroid follicles, forming a major constituent of colloid. Normal thyrocytes show diffuse cytoplasmic staining by TG; this staining pattern is maintained in well-differentiated follicular-derived thyroid carcinomas, such as papillary thyroid carcinoma (PTC) and follicular thyroid carcinoma (FTC), and is completely absent in medullary thyroid carcinoma (MTC) and metastasis to the thyroid gland (**Figure 3**). Focal expression of TG in the follicular component is seen in cases of mixed medullary and follicular thyroid carcinoma. Focal diffuse staining has been reported in >50% of high-grade follicular-cell-derived non-anaplastic carcinomas and is often lost in the foci of necrotic tumor and anaplastic thyroid carcinoma (ATC). In oncocytic lesions, TG usually shows punctate and dot-like perinuclear staining pattern (1, 7, 19–23).

The low or absent production of thyroglobulin by some tumors can lead to diagnostic conundrums, especially in the following clinical scenarios: the use of TG FNA washout evaluation for regional and distant metastasis and the role of serum TG measurement for the follow up of patients with thyroid carcinomas (21, 24, 25).

#### 3.1.3 Thyroid transcription factor 1

Also termed as thyroid-specific enhancer binding protein (NKx2.1), belongs to the family of homologous transcription factors in the NKx2 gene, and is located in the q12–q21 region of chromosome 14. The *TTF-1* gene translates a nuclear protein with an approximate mass of 38 kDa, comprising a single polypeptide of 371 amino acid polypeptides. TTF-1 expression is regulated during embryonic development and appears early in the foregut endoderm and then in the tracheal precursor cells. After birth, TTF-1 expression is confined to the pulmonary type II alveolar cells (26). In the thyroid gland, *TTF-1* expression occurs earlier than the expression of genes related to follicular cell differentiation, such as TG, thyroid peroxidase (TPO), and thyrotropin receptor (TSHR) (27).

TTF-1 shows nuclear expression by IHC in thyroid follicular and parafollicular cells and lung. TTF-1 is diffusely expressed in PTC, FTC, high-grade follicular-derived non-anaplastic thyroid carcinoma and MTC. TTF-1 expression is retained in less than 20% of ATCs (7). TTF-1 is expressed in more than 80% of lung adenocarcinomas, a subset of squamous cell carcinoma of pulmonary origin, small cell carcinoma, neuroendocrine carcinomas, and also rarely in adenocarcinoma of genitourinary and gastrointestinal tracts and breast (26–29).

TTF-1 can be useful in confirming the diagnosis of a thyroid primary lacking a well-differentiated growth pattern (papillary or follicular) or unusual cytology, such as poorly differentiated thyroid carcinoma, mucoepidermoid carcinoma, and secondary tumors (7, 30).

#### 3.1.4 Paired box gene 8

Is a transcription factor that belongs to the paired-box family of genes; it plays a critical role in the development of the thyroid gland, kidney, and Mullerian tract (31). With IHC, its expression is seen in thyroid, renal, and urinary bladder neoplasms and malignancies of Mullerian origin, including ovarian primaries. Several studies have added the following to the repertoire of PAX8 positive tumors: carcinomas of the breast, lung, prostate, gastrointestinal tract, liver and pancreas, testicular tumors, mesothelioma, melanoma, and rhabdomyosarcoma (31–34).

#### 3.1.5 PAX8

Gives a nuclear staining in normal and neoplastic thyrocytes, and usually maintains this expression pattern also in cases of high-grade follicular-cell-derived carcinoma, anaplastic carcinoma, and its squamous subtype (35–37). Among thyroid tumors, a majority of non-follicular cell-derived thyroid carcinomas stain negative for the PAX8 antibody (36). Rare cases of medullary thyroid carcinoma can show PAX8 expression. Intrathyroid thymic carcinoma (ITC) shows a nuclear positive reaction with polyclonal PAX8 antibody but does not react with the monoclonal form. Therefore, monoclonal PAX8 antibody is more specific for thyroid follicular cell origin (36). As noted above, as PAX8 is also expressed in a wide variety of neoplasms from other organs, an initial panel of TTF-1, TG, and PAX8 is needed to confirm or exclude distant metastases from a thyroid primary (35, 37). The other markers used to confirm thyroid follicular cell differentiation include TTF-2 (FOXE1) and thyroid peroxidase (1, 7, 22, 35).

### 3.2 Parafollicular C-cell specific markers

**Figure 4:**
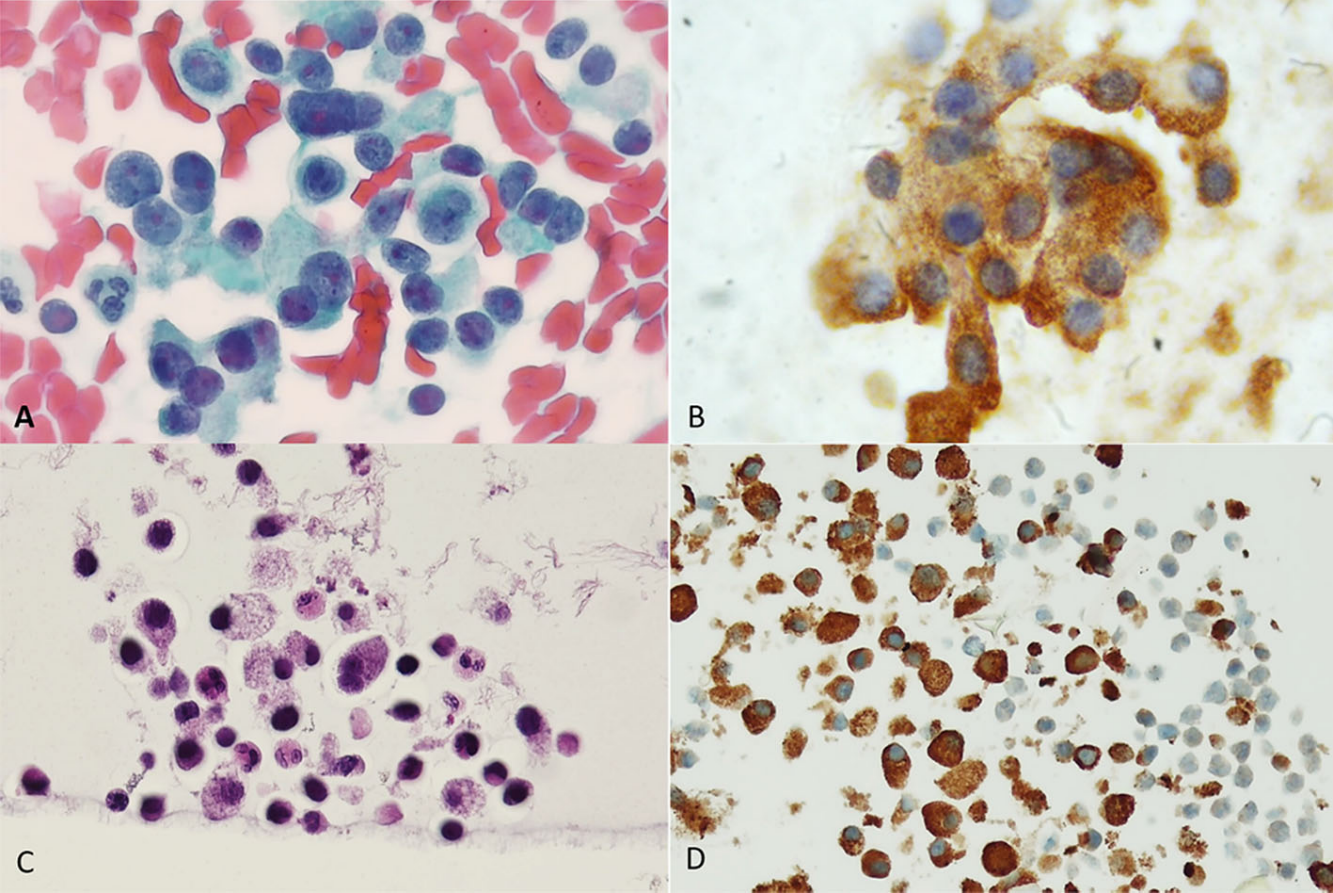
Medullary carcinoma. **(A)** Direct smear. Papanicolaou stain shows a cellular proliferation of polymorphous sometimes plasmacytoid appearing cells with oval nuclei of variable sizes. **(B)** Calcitonin immunostain performed on a destained smear. A granular brown positive reaction is evident in the cytoplasm of the cells. **(C)** Cellblock preparation using the agar gel method. Hematoxylin and Eosin stain shows discohesive cells with granular cytoplasm. Bi-nucleated cells are also evident. **(D)** Calcitonin immunostain shows strong cytoplasmic granular expression.

**Figure 5:**
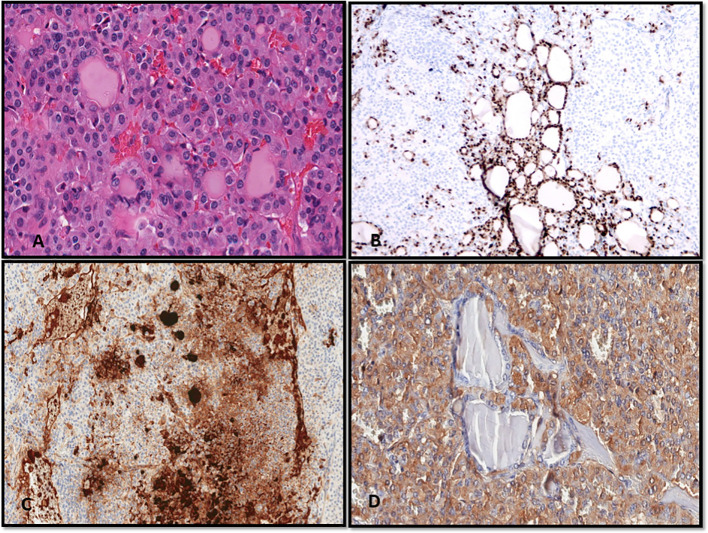
**(A–D)** A case of mixed medullary and follicular carcinoma showing follicle formation with colloid and a solid growth pattern of medullary carcinoma (A, Hematoxylin and Eosin stain). **(B–D)** With immunohistochemistry, the follicular-derived components show expression of TTF-1 **(B)** and thyroglobulin **(C)**; the medullary carcinoma is highlighted by the expression of calcitonin **(D)**.

Medullary thyroid carcinoma (MTC) originates from parafollicular C-cells of the thyroid gland. The C-cells mainly secrete calcitonin hormone, which plays a minor role in calcium metabolism compared with parathyroid hormone (PTH) (38–40). Most MTCs (>95%) secrete calcitonin and show patchy to diffuse cytoplasmic expression of this biomarker with IHC (**Figure 4**). It is well-known that MTC, in addition to its typical nesting growth, tumor cells with nuclear chromatin typical of neuroendocrine tumors (salt and pepper), and amyloid rich tumor stroma, can demonstrate a variety of architectures and cellular features that can be mistaken for other primary thyroid tumors (41–43). In such cases, IHC for calcitonin in pathologic preparations confirms the diagnosis of MTC. This also holds true for rare cases of mixed medullary and follicular thyroid carcinoma, in which calcitonin specifically highlights the MTC components (43) (**Figure 5**).

Owing to its architecture and cellular features, MTC can be difficult to distinguish from metastases to the thyroid from neuroendocrine carcinoma arising in other organs, especially the lung and gastrointestinal tracts (41, 43). It is well known that calcitonin is also expressed in other neuroendocrine tumors besides MTC. In cases in which the diagnostic differential includes MTC and metastatic neuroendocrine carcinoma, clinical correlation and serum calcitonin level, which is often quite increased in MTC, can help determine the correct diagnosis. MTC also shows cytoplasmic expression of monoclonal carcinoembryonic antigen (mCEA), which can also serve as biomarker for disease surveillance in addition to calcitonin (44, 45). This proves to be helpful in rare cases of calcitonin negative MTC (43, 45–47). Additionally, MTC shows expression of other neuroendocrine markers, such as chromogranin, synaptophysin, and rarely CD56. The second-generation neuroendocrine markers insulinoma-associated protein 1 (INSM1), ISL1, and secretagogin show high sensitivity and specificity for neuroendocrine differentiation and maintain the expression even in poorly differentiated neuroendocrine carcinomas. In particular, INSM1 has been reported to be a highly sensitive and specific neuroendocrine marker and is useful in the diagnosis of MTC and C-cell hyperplasia (48–50).

#### 3.2.1 Rare thyroid neoplasms

The value of IHC cannot be underemphasized in the diagnosis of uncommon thyroid neoplasms and those of uncertain histogenesis (1, 2, 51) (see **Table 1**).

**Table 1:** Immunoprofile of key thyroid primary and secondary tumors.

Site of origin	Immunostaining profile*
Thyroid tumors - primary	
1. Follicular cell	CK7+, CK20-, TTF1+, PAX8+, thyroglobulin+
2. C-Cell	CK7+, TTF-1+, PAX8-, calcitonin+, CEA+, synaptophysin+, chromogranin+, thyroglobulin -
Thyroid tumors – primary others	
1. Hyalinizing trabecular tumor	TTF1+, PAX8+, thyroglobulin+, MIB1 (membranous)
2. Mucoepidermoid carcinoma	AE1/AE3+, pan-cytokeratin +, p63+, TTF1**+, PAX8+**, thyroglobulin +**
3. Sclerosing mucoepidermoid carcinoma with eosinophilia	TTF1+, PAX8-, thyroglobulin -
4. Cribriform morular thyroid carcinoma	β-catenin, TTF1+ (mainly in cribriform components), PAX8-, thyroglobulin-,
5. Intrathyroidal thymic tumors	AE1/AE3+, TTF1-, thyroglobulin-, CD5+, p63+, bcl-2+
Parathyroid	TTF1-, PAX8-, thyroglobulin-, calcitonin-, PTH+, GATA3+, chromogranin+
Thyroid tumors - secondary	
1. Pulmonary	CK7+, TTF1+, napsin+, PAX8-, thyroglobulin-
2. Gastrointestinal tract	
I. Esophagus	CK7+, CK20-, TTF-1 -, CDX2 +/-, CEA+, MUC1-/+, MUC5AC -/+, SATB2-
II. Stomach	CK7+, CK20+, TTF-1 -, CEA+, CDX2** MUC1 -/+, MUC5AC-/+
III. Colorectal	CK7-, CK20+, CDX2+, SATB2+, MOC31+
3. Breast	CK7+, CK20-, GATA3+, mammoglobin+/-, GCDFP15-/+, ER+, PR+, TTF-1 -, TG-
4. Melanoma	SOX10+, Melan-A+, S100+, HMB45+, CK7-, CK20-
5. Kidney	
I. Clear cell	CK7-, PAX8+, PAX2+, CAIX+, CD10+, RCC+, AE1/AE3+, CAM5.2+, EMA+, AMACR+/-, GATA3 -, TTF-1-
II. Clear cell papillary	CK7+, PAX8+, CAIX +, CD10-, RCC+/-, AMACR-, GATA3 -/+ (rare cases)
III. Papillary renal cell	CK7+, PAX8+, CA1X+/-, CD10+, RCC+, AMACR +, GATA3 -

*it is preferable that all immunostains should be validated with cytology preparations; **some cases show negative expression; CK, cytokeratin.

## 4 Differentiating benign from malignant thyroid neoplasms

Most thyroid neoplasms are diagnosed based on architectural and cellular features and a lack or presence of invasive features. However, in some instances, it may be difficult to distinguish between follicular adenoma and non-invasive follicular tumor with papillary-like nuclear features (NIFTP), an encapsulated follicular variant of papillary thyroid carcinoma, follicular carcinoma, and follicular adenoma with papillary architecture from papillary thyroid carcinoma.

The diagnosis of follicular carcinoma and the encapsulated follicular variant of papillary thyroid carcinoma requires the evaluation of the tumor-capsule-thyroid interface. Invasion *of* the capsule, invasion *through* the capsule, and invasion *into* veins in or beyond the capsule represent the diagnostic criteria for carcinoma in a follicular-patterned thyroid neoplasm. To this date, what constitutes “*capsular*” invasion in a follicular-patterned thyroid neoplasm remains controversial. Some require penetration *through* the capsule of the tumor and others invasion *into* the capsule to render a diagnosis of either follicular carcinoma or an encapsulated follicular variant of papillary thyroid carcinoma, while others believe that the diagnosis of minimally invasive follicular carcinoma should only be rendered when vascular invasion is present. However, studies have shown that metastatic disease can occur in cases of follicular carcinoma in which only capsular invasion occurred. Thus, “capsular invasion is a sufficient criterion to diagnose malignancy” (1, 2, 4, 6, 52–57).

Despite the controversy regarding capsular invasion as a criterion for malignancy, all agree that angioinvasion is a definite feature of malignancy. It has been shown that encapsulated angioinvasive follicular-patterned tumors carry a significant propensity for clinically malignant behavior. The following histomorphologic criteria have been proposed for the diagnosis of angioinvasion: the invasive tumor should form a plug or polyp in a subendothelial location, enveloping of tumor thrombus by the endothelium, and the tumor thrombus does not have to be attached to the vessel wall to be accepted as an invasion (52–57).

Immunostaining for Factor VIII–related antigen and other endothelial markers, such as CD31, CD34, and ERG, can confirm the foci of angioinavsion. Rarely, histiocytes intermixed with fibrin and inflammatory cells within capsular vessels can mimic foci of angioinvasion. In such instances, macrophage markers, such as CD68 or CD163, and markers for follicular cell lineage, TTF-1, PAX8, and thyroglobulin, can help to confirm the presence of tumor cells within a vessel lumen (52, 53, 55).

The diagnostic conundrum of differentiating a benign from a malignant thyroid lesion is often encountered in limited cellularity fine-needle aspiration (FNA) and core-biopsy specimens. The use of immunohistochemical markers for differentiating benign from malignant thyroid neoplasms in FNA specimens classified as indeterminate is often debated in the literature (11, 12, 58–69).

**Figure 6:**
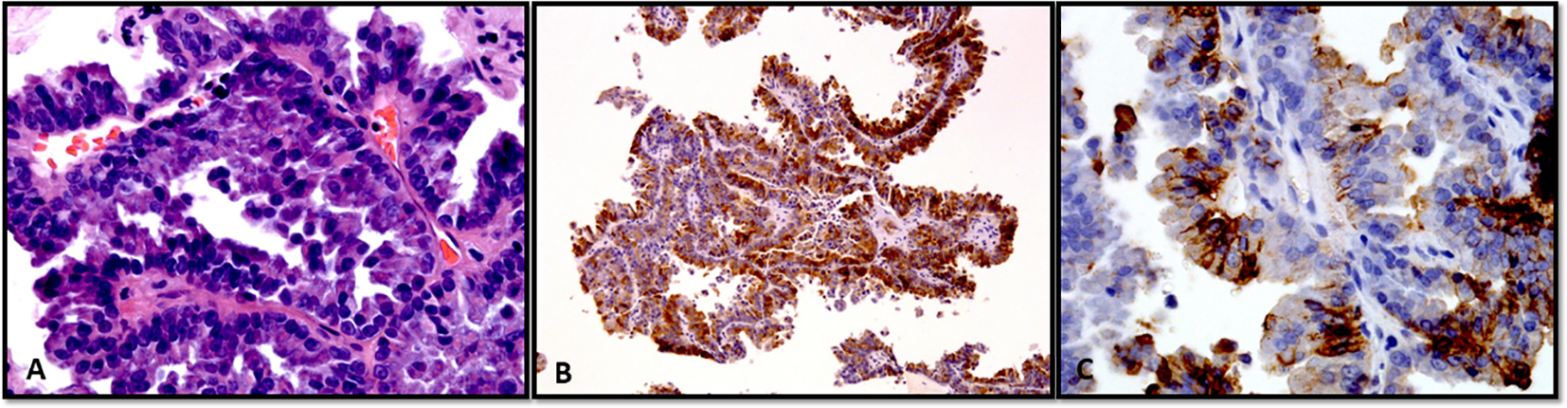
**(A–C)** Fine-needle aspiration cellblock preparation showing a case of papillary thyroid carcinoma. **(A)** Classic subtype (Hematoxylin and Eosin stain). **(B, C)** With immunohistochemistry, the tumor cells show positive expression of thyroglobulin **(B)** and HBME-1 **(C)**.

The combination of HBME-1, GAL-3, and CK19 is by far the most common panel for distinguishing benign from malignant thyroid neoplasms, as no individual biomarker has sufficient sensitivity or specificity to accomplish this task. Combined immunopositivity for Gal-3, CK19, and HBME-1 shows high sensitivity (95%) and specificity (97%) for the diagnosis of papillary thyroid carcinoma (11, 12, 58–64, 66, 68, 69) (**Figure 6**). Combined immunoexpression of Gal-3 and CK19 had 92% sensitivity and 99% specificity while combined positivity for Gal-3 and HBME-1 had 95% sensitivity and 95% specificity for papillary thyroid carcinoma (64). It should be noted that the expression of HBME-1, GAL-3, and CK19 is not predictive of the clinical aggressiveness of the tumor and cannot be used to guide the surgical excision (64).

Galectin 3 (Gal-3) has received significant attention for its utility as a diagnostic marker for thyroid cancer, being positive in thyroid carcinoma and negative in benign neoplasms and normal thyroid tissue (70). In a meta-analysis of 8,172 thyroid nodules with histologic evaluation, Gal-3 IHC was reported to be positive in 87% of thyroid cancers, confirmed by histopathologic follow-up. This information confirms that many thyroid carcinomas have overexpression of this marker. A Gal-3 test on thyroid FNA samples (cellblock preparation) has a sensitivity lower than that observed in histologic preparations (pooled histologic sensitivity of Gal-3 was 96%, while sensitivity with FNA was 90%); mainly due to the different methods used for Gal-3 evaluation in thyroid cytological specimens, technical variability in antibody clones and immunostaining protocols, and relevant differences in staining interpretation (i.e. nuclear, cytoplasmic, or membranous positivity) (12). In summary, the use of Gal-3 in cellblock preparation from thyroid FNA may support a diagnosis of malignancy in thyroid nodules classified as indeterminate. In addition to galectin-3, other markers such as Hector Battifora mesothelial cell-1 (HBME-1), cytokeratin-19 (CK19), and cluster differentiation antigen 56 (CD56) can facilitate the diagnosis of thyroid carcinoma in both histologic and cytologic preparations (62–64, 71) (**Figure 6**).

Ki-67 is the protein product of the gene *MKI67* and is a commonly used IHC marker for cell proliferation. Recently, the Ki-67 index has been proposed for the stratification of PTC, FTC, and MTC into different risk categories. The proposed Ki-67 indices show that differentiated thyroid carcinomas can be stratified into low-, moderate-, and high-risk groups using the cutoff values of <5%, 5–10%, and 10–30% (72–75). A two-tiered system is recommended for Ki67 evaluation in medullary thyroid carcinoma, employing a cutoff of 5% (76).

With light microscopy, at least one of three features, mitotic index of ≥5 per 2 mm^2^, Ki67 proliferative index of ≥5%, or tumor necrosis, is required to define high-grade MTCs, and these criteria have been integrated in the 5th edition of the World Health Organization classification of thyroid tumors (1, 2). Additionally, the use of Ki67 IHC with cytological samples from thyroid FNA has been investigated. In a recent study, the authors applied a scoring evaluation for calculating the percentage of positive cells by counting at least 200 tumor cells; they concluded that the Ki-67 index determined in cytology specimens significantly correlates with the Ki-67 index obtained by immunohistochemical analyses of histologic specimens (77). This analysis was performed on air-dried smears that were formalin fixed before immunostaining.

IgG4-thyroid-related disease (TRD), although uncommon, is a spectrum of diseases with a clinical presentation that can often mimic malignancy. The threshold to confirm increased IgG4-positive plasma cells ranges from more than 20 to more than 30 IgG4-positive plasma cells per high-power field by microscopic examination (78–81). The FNA specimens show lymphoplasmacytic infiltrates and oncocytes and the cytological features are usually classified as benign (80). Clinical history, radiological characteristics, and cytological features, such as abundant plasma cells, fibroblast, and epithelial atypia, should raise the suspicion of IgG4-related disease (82). If IgG4 TRD is clinically suspected at the time of FNA, IHC might confirm the predominance of IgG4-secreting plasma cells in the cytological sample, leading to additional clinical workup.

## 5 Molecular immunohistochemistry

Molecular profiling of thyroid carcinomas with aggressive clinical behavior has become a standard of care. Modern immunohistochemistry has proven to be an easily practiced approach in the everyday practice of histopathology to triage advanced tumors for further mutation testing. Specific IHC is available for BRAFV600E mutation, RASQ61R mutation, NTRK rearrangement, and ALK rearrangement. Of note, among the IHC for these altered proteins derived from molecular changes, only IHC for BRAFV600E is approved to be of value in the clinical management of malignant thyroid neoplasms; other mutation-specific IHC only confirms the presence or absence of mutation or rearrangement (83). IHC using mutation-specific antibodies against BRAFV600E (VE1 clone, Spring Bioscience, Pleasanton, CA) provides an alternative inexpensive method for the rapid identification of BRAFV600E mutation-positive thyroid tumors. The overall reported sensitivity and specificity of *BRAF p.V600E* immunostaining with cellblock preparation is 94.4% and 100%, respectively; however, this approach is not recommended for FNA smears and monolayer preparations (84).

Consensus guidelines drawn up by an international expert panel do not recommend IHC for NTRK fusion confirmation; however, in some cases, IHC can be used for preliminary screening (85). Similarly, ALK IHC is suggested as a screening procedure, and FISH analysis is recommended for the final confirmation of ALK rearrangement (86). The IHC for RET should not be considered as an option for pre-screening (71).

IHC also allows the characterization of tumor microenvironment (PD-L1 and CD markers) and its role in predicting the response of thyroid cancer to immunotherapy (13). Different scoring systems for PD-L1 immunostaining have been approved by the FDA as companion diagnostic tests for patient selection for the treatment of various tumors, such as melanoma and lung cancer. PD-L1 expression in thyroid cancer has been shown to be similar to that in other solid tumors. A study of 407 primary thyroid cancers showed PD-L1 expression in 6.1% of papillary thyroid carcinomas, 7.6% of follicular thyroid carcinomas, and 22.2% of anaplastic thyroid carcinomas at a threshold of 1% (87). In a recent meta-analysis, the frequency of PD-L1 positivity in thyroid tumor cells for different histological types ranged from 7% to 90%. This study also demonstrates the role of PD-L1 expression as a potential prognostic marker of disease recurrence in patients with papillary thyroid carcinoma (88).

The clinical utility of determining PD-L1 expression supports the use of PD-L1 immunotherapy as a part of combination therapy in metastatic and RAI-refractory thyroid cancer (89). The optimal cutoff value for immunohistochemical positivity of PD-L1 immunostaining has not yet been validated in thyroid cancer, and the variability in different studies depends on the selected clone, the immunostaining method, and the morphological interpretation. When PD-L1 immunoreaction is used for treatment purposes, it is mandatory that only membranous staining of PD-L1 is considered positive and not the cytoplasmic expression (13).

A recent review has shown that cytological samples constitute a reliable source for PDL-1 IHC analysis, as evidenced by the tumor-rich specimens and concordant results between cytological and histological specimens (90). This study emphasizes that the fixatives used in today’s cytology laboratories do not compromise PD-L1 staining, attesting to the utility of cytological specimens for PD-L1 testing in routine clinical practice. PD-L1 IHC may predict the success of PD-1 blockade therapy in a subset of patients with an anaplastic carcinoma PD-1 tumor proportion score of >=1% (91).

### 5.1 Technical considerations for immunohistochemistry

**Figure 7:**
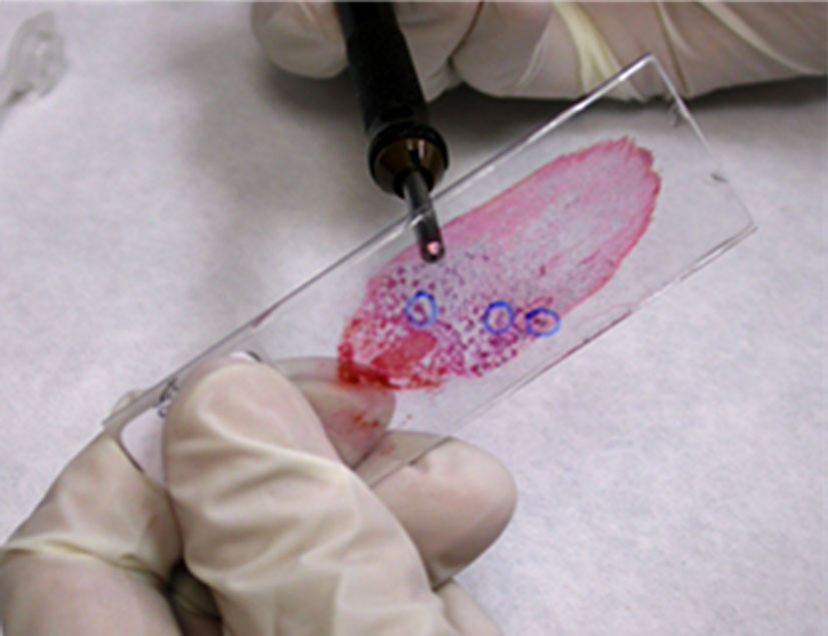
A smeared slide is prepared for destaining and treatment for immunocytochemistry. The cell groups of interest are circled by a pen on the coverslip under the microscope as reference, and then they are circled with a glass etching pen on the back of the slide. This step will make it easier to identify the lesional cells after immunostaining.

Destained smear slides prepared during the rapid onsite evaluation of FNA specimens have been used for IHC in cytology. This technique only allows the use of either one or two immunostains and helps the characterization of the cell of origin (follicular, parafollicular/C-cells, metastatic disease, and hematologic neoplasms). In specimens with limited cellularity, areas of interest on the smear can be circled with a glass pen on the reverse slide to easily locate the cell cluster after immunostaining (**Figure 7**). As a general rule, international guidelines recommend that cellblock is the cytology preparation of choice for performing a panel of immunostains (15, 92).

Cytology cell blocks are made by employing different fixatives and paraffin to obtain morphology similar to histologic preparations and can be routinely used for IHC. Different techniques can be used to prepare cellblocks either by automatic instruments or ready to use gels and matrices. Quality control assessment for immunostaining with cytological samples is a mandatory requirement for each laboratory. UK NEQAS ICC for the quality of immunocytochemical staining reported that cellblock sections achieved the highest score (93). Cellblock preparations are also recommended for “molecular immunohistochemistry”, in which markers are designed to recognize the presence of altered proteins from mutated genes (71, 85, 94).

## 6 Conclusion

Currently, immunohistochemical and molecular analysis are integral to the diagnosis and management of thyroid neoplasms. Accurate diagnosis and classification of thyroid tumors according to the recent classification scheme can be achieved by employing specific immunostains in both histologic and cytologic specimens (1). New multiplex chromogenic and multiplex fluorescent IHC are emerging technologies that enable the simultaneous detection of multiple biomarkers in a single tissue section. Their development for preclinical research and clinical application has increased extraordinarily in the last 5 years, paving the way for a better understanding of tumorigenesis and clinical behavior, and they are expected to improve the personalized treatment of patients with malignant tumors of the thyroid gland (95).

## Author contributions

All authors listed have made a substantial, direct, and intellectual contribution to the work and approved it for publication.
